# Early hearing detection and intervention: Reflections from the South African context

**DOI:** 10.4102/sajcd.v65i1.581

**Published:** 2018-04-19

**Authors:** Amisha Kanji

**Affiliations:** 1 Department of Speech Pathology and Audiology, University of the Witwatersrand, South Africa

## Abstract

For researchers and clinicians in developing contexts like South Africa, the establishment of universal newborn hearing screening (UNHS) programmes is something which we have strived to achieve. However, we need to ask the question as to whether we have attempted to view our ultimate goal of achieving mandated UNHS programmes from the perspective of the South African healthcare system as a whole. The current manuscript is aimed at providing an overview of audiological services within a broader context, with reflections from a South African perspective, and a suggestion to consider alternatives to UNHS, particularly in the South African public health care sector.

## The need for early hearing detection and intervention

There is a convincing argument, and enough published research evidence, for the need for early detection of hearing loss in newborns and infants, with universal newborn hearing screening (UNHS) being the gold standard. Undoubtedly, there is enough evidence that proves the positive outcomes and benefits that result from early hearing detection and intervention (EHDI), particularly UNHS (Fulcher, Purcell, Baker, & Munro, 2012; Kennedy et al., 2006; Pimperton & Kennedy, 2012). This evidence mainly stems from developed contexts where EHDI programmes have been mandated or are established and well run, even with trained screening personnel (McPherson, 2012; Singh, 2015).

## Striving towards universal newborn hearing screening

For researchers and clinicians in developing contexts like South Africa, the establishment of UNHS programmes is something which we have strived to achieve. Firstly, this is partly because we tend to use current research from the developed world to justify evidence-based best practice in each of our contexts. Secondly, it is because we want to achieve the goals outlined in position documents from our regulatory bodies. Whilst these serve as good reasons to aim towards best practice from the developed contexts (which form the benchmark), there is a sense that we are working within our individual departments and in silos within the bigger picture of the health care system in our context. The lack of efficient data management systems for EHDI, which are largely paper based and do not allow for sharing of information with other professionals adds to this complex context (Moodley & Storbeck, 2017). There have been some attempts at exploring newborn hearing screening (NHS) within the South African context, which have included a single study related to UNHS within a private health care hospital in Gauteng, and a few studies related to the implementation of UNHS at primary health care (PHC) clinics and Midwife Obstetric Units in Gauteng and the Western Cape (de Kock, Swanepoel, & Hall, 2016; Khoza-Shangase & Harbinson, 2015; Swanepoel, Ebrahim, Joseph, & Friedland, 2007). Whilst these studies have informed the implementation of NHS in a contexts, they are limited to a few provinces only, and findings have not in all instances led to the actual implementation of fully fledged national programmes. Have we paused and attempted to view our ultimate goal of achieving mandated UNHS programmes from the position of the South African healthcare system as a whole?

## Considering early hearing detection and intervention within the broader South African health care system

Health should be considered within the broader context of links (direct and indirect) between health and wealth (Mayosi & Benatar, 2014). Our current healthcare in South Africa consists of a large public health care sector which is funded by the state. The majority (84%) of the population is dependent on this public health care system (Naidoo, 2012), which is served by only 30% of health care professionals (DoH, 2011; van Rensburg, 2014). Whilst the distribution of the number of births and the population accessing public versus private health care is similar in some developed countries such as the United Kingdom, their health sector had the largest number of employees in 2012, and the number of health care workers within their national health service has grown over the years (Jayaweera, 2015).

Unlike countries such as the United Kingdom where health systems function relatively well with less money being spent on health as a percentage of GDP, 40% of all expenditure for health in South Africa comes from National Treasury, with public health consuming 11% of the total government budget (Cylus et al., 2015; Jobson, 2015). These figures are higher than the 5% of GDP recommended by the World Health Organization (WHO) and reflect the huge burden of disease requiring management within the public health care sector (Jobson, 2015). This high level of burden of disease highlights the need for an increase in the number of health care professionals; yet, we need to be mindful that quality of life often directly links to staying alive (Mayosi & Benatar, 2014; Moyakhe, 2014), with little or no focus on impairments such as those related to hearing and communication.

With over 1 million births per year in South Africa (Statistics South Africa, 2016), and an estimated 6116 of these babies presenting with congenital, permanent or acquired hearing loss within the first few weeks of life (Swanepoel, 2009), there is a clear need for sufficient staffing of audiologists. However, the capacity: demand ratio may be far from ideal with an overall 5216 registered speech pathology and audiology professionals in South Africa (HPCSA, 2017). Although this figure has increased slightly from 4892 in 2016, there were only 503 registered audiologists and 1510 dually qualified speech pathologists and audiologists (HPCSA, 2016), which poses a serious manpower challenge towards the implementation of UNHS services. This is in comparison with other developed countries such as Australia and the United States where overall health care workforce may be large (Jayaweera, 2015) and/or where NHS services are well established and conducted by other healthcare professionals, audiology technicians or trained volunteers other than audiologists (Choo & Meinzen-Derr, 2010; Ferro, Tanner, Erler, Erickson, & Dhar, 2007; Olusanya, 2011; WHO, 2010), possibly contributing towards the overall feasibility and success of UNHS programmes. In other developing countries, such as Lagos, Nigeria, the use of non-specialists and community healthcare workers as screeners (at an inner city environment and immunisation clinics, respectively) has been reported to be feasible in effecting NHS services (Olusanya, Wirz, & Luxon, 2008). However, screening by non-specialists would require the ongoing need for staff training provision and maintenance of equipment. Furthermore, the Health Professions Council of South Africa has regulations governing the provision of health care, and the use of such cadres of screeners would need to be regulated by such a governing body.

## Recommendations and considerations for South Africa

Considering the realities of the South African health care context, and given that EHDI is vital for newborns and infants with hearing loss, we need to seriously consider how NHS services may be adapted to better meet these realities. One option is to seriously consider targeted NHS as a starting point or interim approach to early identification, particularly within a hospital setting (Kanji, 2016). The reason being that certain medical case history factors may predispose or place newborns at a greater risk for significant hearing loss. Newborns or infants with these medical risk factors may be more likely to be admitted or referred to a hospital setting (McPherson, 2012). Other risk factors such as family history of hearing loss and Human Immunodeficiency Virus (HIV) exposure (HPCSA, 2007) may result in admission to well-baby nurseries within the hospital context, and these newborns and infants should therefore be monitored at their first follow-up visit at the relevant health care context.

Efforts to achieve sustainable improvements in health in the context of limited resources suggests the need for improvement in healthcare management and extensive shifts in attitude to service to ‘doing better with less’ (Mayosi & Benatar, 2014, p. 1351). It is this position that has led me to reflect on South Africa’s engagement with EHDI, how we have progressed, and if not, whether we are failing to be ‘doing better with less’. Having framed EHDI within the broader context of health in South Africa, and through my research and clinical engagements in the field, I have asked the following questions: Why UNHS and why now? Why is targeted NHS not considered a sufficient, realistic, interim solution and a good stepping stone to achieving quality audiological services within existing resources, with the aim of perfecting these services whilst striving towards the international gold standard? Perhaps we need to consider the following aspects as recommendations in an attempt to answer these questions and approach the issue of EHDI services in South Africa:

- factors contributing towards the reluctance to provide the best of services within our means
- our agenda as audiologists and what we convey towards achieving successful NHS programmes within the EHDI framework
- our actual achievements within the EHDI framework
- the EHDI goals that are truly actionable at this very point in time in our context.

To achieve ‘doing better with less’ as a profession in South Africa, what should we be advocating as *standard* care in the quest for the *ideal?* Are we attempting to digest the elephant as a whole instead of piecemeal as a logical step?

All programmes need to start somewhere and undergo their infancy stages. Hence, perhaps it is time that we reflect and realise that conducting targeted or risk-based NHS is in fact a good foundation for the infancy stages and that doing something well is better than doing nothing at all, particularly in the public health care sector which is under-resourced. We also need to realise that effective and quality healthcare is not only dependent on individual professionals but involves other main stakeholders such as the government and civil organisations (Moyakhe, 2014). As health care professionals we need to acknowledge the limitations but not allow it to preclude us from providing quality services within our means.
